# Adaptive landscape genetics and malaria across divergent island bird populations

**DOI:** 10.1002/ece3.5700

**Published:** 2019-10-02

**Authors:** Claire Armstrong, Richard G. Davies, Catalina González‐Quevedo, Molly Dunne, Lewis G. Spurgin, David S. Richardson

**Affiliations:** ^1^ School of Biological Sciences University of East Anglia Norwich UK; ^2^ Grupo Ecología y Evolución de Vertebrados Instituto de Biología Facultad de Ciencias Exactas y Naturales Universidad de Antioquia Medellín Colombia

**Keywords:** birds, genome-wide association studies, Haemosporidians, landscape genetics, malaria, pathogen‐mediated selection, Toll‐like receptor

## Abstract

Environmental conditions play a major role in shaping the spatial distributions of pathogens, which in turn can drive local adaptation and divergence in host genetic diversity. Haemosporidians, such as *Plasmodium* (malaria), are a strong selective force, impacting survival and fitness of hosts, with geographic distributions largely determined by habitat suitability for their insect vectors. Here, we have tested whether patterns of fine‐scale local adaptation to malaria are replicated across discrete, ecologically differing island populations of Berthelot's pipits *Anthus berthelotii*. We sequenced TLR4, an innate immunity gene that is potentially under positive selection in Berthelot's pipits, and two SNPs previously identified as being associated with malaria infection in a genome‐wide association study (GWAS) in Berthelot's pipits in the Canary Islands. We determined the environmental predictors of malaria infection, using these to estimate variation in malaria risk on Porto Santo, and found some congruence with previously identified environmental risk factors on Tenerife. We also found a negative association between malaria infection and a TLR4 variant in Tenerife. In contrast, one of the GWAS SNPs showed an association with malaria risk in Porto Santo, but in the opposite direction to that found in the Canary Islands GWAS. Together, these findings suggest that disease‐driven local adaptation may be an important factor in shaping variation among island populations.

## Graphical abstract

We have investigated patterns of fine‐scale local adaptation to malaria between two ecologically divergent populations of an island bird. Our results found contrasting associations with malaria infection at candidate immune loci between the islands, suggesting that disease‐driven local adaptation may be an important factor in shaping variation among island populations.

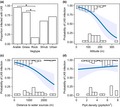

## 1 INTRODUCTION

Spatial variation in the environment is a key force driving local adaptation and population divergence (Hereford, 2009). There is increasing evidence that fine‐scale changes in environmental conditions can result in variation in selection pressures over small geographic distances (Garroway et al., 2013; Langin et al., 2015; Richardson, Urban, Bolnick, & Skelly, 2014) and that fine‐scale adaptation can persist despite the homogenizing effects of gene flow (e.g., Lenormand, 2002). Adaptation to spatially heterogeneous environmental conditions can also facilitate balancing selection, where genetic variation is maintained within and between populations due to differential selection on genetic variants (Bockelmann, Reusch, Bijlsma, & Bakker, 2003; Levene, 1953; Schmidt, Bertness, & Rand, 2000).

Pathogens are major drivers of evolution, exerting strong selective pressures on their hosts (Fumagalli et al., 2011). The high genetic variation found at many genes involved in immune processes is thought to be maintained by pathogen‐mediated balancing selection (Bernatchez & Landry, 2003; Ferrer‐Admetlla et al., 2008; Spurgin & Richardson, 2010). Spatial variation in pathogen‐mediated selection has the potential to drive fine‐scale heterogeneity in immunogenetic diversity (Larson, Lisi, Seeb, Seeb, & Schindler, 2016; Tschirren, Andersson, Scherman, Westerdahl, & Råberg, 2011), highlighting the importance of spatial scale in understanding pathogen‐mediated selection.

Haemosporidians in the genera *Plasmodium*, *Haemoproteus*, and *Leucocytozoon* (hereafter termed malaria for simplicity) are protozoan parasites that infect the red blood cells of mammals, reptiles, and birds (Martinsen, Perkins, & Schall, 2008). Infection by malaria has been associated with increased mortality, decreased body condition, and reductions in fitness (Guggisberg, Sayler, Wisely, & Odom John, 2018; Knowles, Palinauskas, & Sheldon, 2010; Marzal, Bensch, Reviriego, Balbontin, & De Lope, 2008). The selective pressure exerted by malaria has driven the evolution of increased host resistance and tolerance (Atkinson, Saili, Utzurrum, & Jarvi, 2013; Hill et al., 1991), with evidence of local adaptation to spatially heterogeneous selection pressures (Loiseau et al., 2011; Piel et al., 2010). Malaria parasites are dependent on vector transmission to complete their lifecycle, and their spatial distributions are therefore constrained by the environmental niches of their insect vectors. Temperature, rainfall, and altitude play especially important roles in determining malaria prevalence (Illera, López, García‐Padilla, & Moreno, 2017; Jones, Cheviron, & Carling, 2013; Padilla, Illera, González‐Quevedo, Villalba, & Richardson, 2017). Water is essential for aquatic larval development of vectors, and topographic features that increase surface water persistence promote increased abundance (Ferraguti et al., 2016; Ganser et al., 2016; González‐Quevedo, Davies, & Richardson, 2014). In addition, anthropogenic factors such as habitat degradation, agriculture, and urbanization can all influence malaria dynamics (González‐Quevedo et al., 2014; Turcotte, Bélisle, Pelletier, & Garant, 2018; Yanoviak, Paredes, Lounibos, & Weaver, 2006). Together, these factors have the potential to shape fine‐scale spatial structuring in pathogen selection pressures and host immunogenetic variation.

Studying pathogen‐mediated selection has largely involved a candidate gene approach, where variation at genes with known, or predicted, host immunity function is investigated in relation to infection (Bernatchez & Landry, 2003; Netea, Wijmenga, & O'Neill, 2012). Many studies have focused on the major histocompatibility complex (MHC), a gene family that plays a key role in pathogen recognition in the adaptive immune system. However, a greater proportion of phenotypic variance in malaria response has been attributed to non‐MHC genes (Jepson et al., 1997). Within the innate immune system (a first line of defense against infection), Toll‐like receptors (TLRs) are a family of pattern‐recognition receptors which have been linked to malaria resistance (Ferwerda et al., 2007; Mockenhaupt et al., 2006), and show evidence of pathogen‐mediated balancing selection (Ferrer‐Admetlla et al., 2008; Fisher et al., 2011; Gavan, Oliver, Douglas, & Piertney, 2015). TLRs therefore represent important candidates for investigating the role of pathogens in maintaining host genetic variation.

An alternative to the candidate gene approach is the use of genome‐wide association studies (GWAS). These enable detection of single nucleotide polymorphisms (SNPs) throughout the genome that show statistical associations with pathogen infection. In addition to identifying associations at known immune loci (Fellay et al., 2007; He et al., 2015; Wong et al., 2010), GWAS approaches may reveal novel candidate genes (Fu et al., 2012; Ravenhall et al., 2018; Thye et al., 2010) for further study of the evolutionary dynamics between host and pathogen.

Islands are excellent environments for investigating pathogen‐mediated selection. In line with island biogeography theory (MacArthur & Wilson, 1967), pathogen diversity and abundance are often lower on islands compared with the mainland, simplifying the study of host–pathogen interactions (Pérez‐Rodríguez, Ramírez, Richardson, & Pérez‐Tris, 2013; Clark, Clegg, & Lima, 2014; but see Illera, Fernández‐Álvarez, Hernández‐Flores, & Foronda, 2015). Pathogen communities on each island are shaped by chance colonization and extinction events, which can result in distinct pathogen assemblages and selection pressures between islands (Fallon, Bermingham, & Ricklefs, 2005; Olsson‐Pons, Clark, Ishtiaq, & Clegg, 2015; Wang et al., 2017). Limited gene flow in and out of islands also allows for stable communities of hosts and pathogens (Spurgin, Illera, Padilla, & Richardson, 2012), which may facilitate strong coevolutionary relationships.

**Figure 1:**
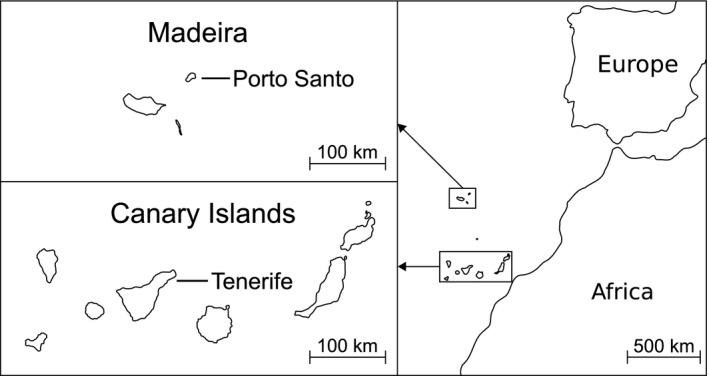
Map of Berthelot's pipit populations. Berthelot's pipits are found across all islands within the Madeiran archipelago (top left panel), the Canary Islands (bottom left panel), and the Selvagens archipelago, situated between the Canary Islands and Madeira

Berthelot's pipit *Anthus berthelotii* is a small sedentary passerine endemic to three Macaronesian archipelagos (Figure 1). Following the colonization of the Madeiran archipelago from the Canary Islands ca. 8,500 years ago (Spurgin, Illera, Jorgensen, Dawson, & Richardson, 2014; but see Valente et al., 2017), there has been a lack of gene flow between the archipelagos (Illera, Emerson, & Richardson, 2007; Spurgin et al., 2014), potentially facilitating local adaptation and divergent selection (Armstrong et al., 2018). Malaria infection shows high spatial variability in this species, both between and within islands, making it a highly suitable model for investigating the role of spatial scale in pathogen‐mediated selection. Characterization of malaria throughout Berthelot's pipit populations (Illera, Emerson, & Richardson, 2008; Spurgin et al., 2012) found the highest prevalence of *Plasmodium* and *Leucocytozoon* infection on Porto Santo, whereas no infection was detected elsewhere in the Madeiran archipelago. Prevalence of malaria on Tenerife is influenced by a combination of climatic and anthropogenic effects (González‐Quevedo et al., 2014; Padilla et al., 2017), with malaria undetected at high altitude. Associations between the distribution of MHC variants and environmental predictors of malaria infection have been detected (González‐Quevedo, Davies, Phillips, Spurgin, & Richardson, 2016). No evidence of *Haemoproteus* infection has been found in this species.

Here, we test for associations between fine‐scale patterns of genetic variation and malaria in Berthelot's pipits across two divergent populations (Tenerife and Porto Santo) to investigate the spatial scale of local adaptation in the presence of gene flow within a population. This study also allows us to test the repeatability of patterns of association across populations. These two islands, situated on different archipelagos, show high genetic divergence at neutral loci, with limited to no gene flow between them (Armstrong et al., 2018; Spurgin et al., 2014). Despite a sharp decline in overall genetic diversity associated with the initial colonization of Madeira (Armstrong et al., 2018), higher levels of TLR4 allelic and amino acid richness exist in Madeira compared to the Canary Islands (González‐Quevedo, Spurgin, Illera, & Richardson, 2015). Furthermore, evidence of positive selection at TLR4 in Berthelot's pipits suggests it may be an evolutionarily important locus (González‐Quevedo et al., 2015). In this study, we (a) test for associations between *Plasmodium* infection status (the only Haemosporidian genus commonly detected; >1% prevalence) in this species (Spurgin et al., 2012), and variation at TLR4 and two SNPs previously identified in a GWAS of malaria infection across Berthelot's pipit populations (Armstrong et al., 2018); (b) determine the environmental predictors of malaria risk on Porto Santo; (c) compare genetic associations with malaria risk in Porto Santo and Tenerife, utilizing the above measures of malaria risk for Porto Santo, and those previously calculated for Tenerife (González‐Quevedo et al., 2016, 2014).

## 2 MATERIALS AND METHODS

### 2.1 Sample collection

Berthelot's pipits were sampled on Tenerife (Feb–April 2006, Jan–Aug 2009, April–May 2010, Jan–May 2011) and Porto Santo (Sept 2006, March 2009, April–June 2016). For samples collected prior to 2011, 30–96 birds were sampled widely across each island per season (Illera et al., 2007; Spurgin et al., 2012). For samples collected in Tenerife in 2011, attempts were made to catch one pipit in each 1 km^2^ of suitable habitat, with a total of 388 birds sampled (González‐Quevedo et al., 2014). On Porto Santo in 2016, all areas of suitable and accessible pipit habitat throughout the island were surveyed. We attempted to catch every pipit encountered, resulting in a sample of 129 birds. In total, 780 birds were sampled across all years and islands.

Birds were caught in spring traps baited with *Tenebrio molitor* larvae. Each bird was fitted with a colored plastic ring or a numbered aluminum ring issued by the Spanish or Portuguese authorities as appropriate, to avoid resampling individuals. Blood samples (ca. 50 μl) were collected by brachial venipuncture and stored in 100% ethanol in a screw‐top Eppendorf tube at room temperature. Birds were classified as juvenile or adult based on feather molt pattern (Cramp, 1988).

### 2.2 Molecular methods

DNA was extracted using a salt extraction protocol (Richardson, Jury, Blaakmeer, Komdeur, & Burke, 2001). Sexing PCRs (Griffiths, Double, Orr, & Dawson, 1998) determined the sex of the bird and confirmed that DNA extractions were successful.

### 2.3 Parasite screening

We used a nested PCR approach that detects *Plasmodium* and *Haemoproteus* to characterize malaria infection status (Waldenström, Bensch, Hasselquist, & Östman, 2004), with multiple positive and negative controls included in each PCR plate. Samples that successfully amplified at least twice were classified as infected. The strain of *Plasmodium* was determined by Sanger sequencing; *Haemoproteus* was not detected. We focused on *Plasmodium* as the most widespread and abundant haemosporidian found in Berthelot's pipits (Illera et al., 2008; Spurgin et al., 2012). In addition, the vector species of *Plasmodium* (mosquitoes; Culicidae) and *Leucocytozoon* (blackflies; Simuliidae) have different ecological niches (Harrigan et al., 2014; Imura et al., 2012), thus combining the two may confound results.

### 2.4 Sanger sequencing and SNP genotyping

A previous TLR4 study genotyped 23–30 individuals from all 13 Berthelot's pipit populations, including Tenerife and Porto Santo (González‐Quevedo et al., 2015). The primers *PauTLR4F*, *PauTLR4R* (Grueber, Wallis, King, & Jamieson, 2012) were used to amplify a 660‐bp region located within the leucine‐rich repeat domain of TLR4. Five SNPs were found: TLR4_1 (905 bp, nonsynonymous), TLR4_2 (970 bp, nonsynonymous), TLR4_3 (990 bp, synonymous), TLR4_4 (992 bp, nonsynonymous, triallelic), and TLR4_5 (1,010 bp, nonsynonymous). Base‐pair positions are stated according to the zebra finch TLR4 protein coding region, GenBank accession FJ695612. Further Sanger sequencing of TLR4 was performed on all samples collected from Porto Santo in 2016 (*n* = 129). TLR4 SNPs were called through visual inspection of chromatograms in FinchTV (https://digitalworldbiology.com/FinchTV). We used LGC Genomics' proprietary KASP™ genotyping technology (https://www.lgcgroup.com) to genotype all additional samples from Tenerife and Porto Santo (*n* = 577) at each TLR4 SNP, except for TLR4_5 which was excluded due to a very low minor allele frequency of <0.05. Assay design and genotyping were performed by LGC Genomics, Hertfordshire.

A GWAS performed on restriction site‐associated DNA sequencing (RAD‐seq) data from Berthelot's pipits by Armstrong et al. (2018) detected two SNPs (5239s1, Chr10:12048280; 7259s1, Chr20:6483195; SNP positions on zebra finch genome v3.2.4, Warren et al., 2010) that showed significant associations with *Plasmodium* strain LK6 (Ortego, Calabuig, Cordero, & Aparicio, 2007) infection. All birds were genotyped with KASP™ assays at these two SNPs.

### 2.5 Genetic analysis

Genotypes AT, CT, and TT at the triallelic TLR4_4 SNP were coded as missing data (Tenerife *n* = 12; Porto Santo *n* = 1), to treat this SNP as biallelic. We used DnaSP v6 (Librado & Rozas, 2009) to phase the TLR4 SNPs into haplotypes. To aid phasing, we included TLR4 sequences from all Berthelot's pipit populations, and each phased TLR4 haplotype previously detected in Berthelot's pipits (González‐Quevedo et al., 2015). Samples with <90% phasing certainty were excluded from models that included TLR4 haplotypes as predictors. We translated the phased TLR4 sequences originating from Sanger sequencing into protein haplotypes. This gave us the amino acid residues at each of the codons containing a SNP, from which we were able to infer the amino acids at each SNP for samples that were genotyped with KASP™ genotyping.

We used PLINK 1.9 (Chang et al., 2015) to calculate linkage disequilibrium (LD) between each pair of SNPs, and test for deviations from Hardy–Weinberg equilibrium with Hardy–Weinberg exact tests. Where frequencies ≤0.05 were found for SNP minor alleles or TLR4 protein haplotypes, these variants were not included as predictors in genetic analyses.

### 2.6 Porto Santo GIS analyses

A previous study demonstrated the importance of environmental and anthropogenic factors in shaping malaria risk in Tenerife (González‐Quevedo et al., 2014). Variables were selected based on the potential for influencing the abundance of avian malaria or its mosquito vectors: the minimum temperature of the coldest month (MINTEMP), annual precipitation (PRECIPITATION), altitude (ALTITUDE), aspect (ASPECT), slope (SLOPE), pipit density (DENSITY), vegetation type (VEGTYPE), distance to nearest poultry farm (DISTPOUL), distance to nearest livestock farm (DISTFARM), distance to nearest artificial water reservoir (DISTWATER), and distance to urban site (DIST_URB). We calculated the values of these variables at each sampling location on Porto Santo as outlined below.

All GIS analyses were performed in QGIS v2.18 (QGIS Development Team, 2017). MINTEMP and PRECIPITATION were obtained from WorldClim global climate data v2 (Fick & Hijmans, 2017) at a resolution of 30 arc‐seconds (approximately 1 km^2^). ALTITUDE was obtained from the Shuttle Radar Topography Mission (SRTM) 3 Arc‐Second Global elevation data (http://srtm.csi.cgiar.org) at a resolution of approximately 90 m^2^. SLOPE and ASPECT were calculated from SRTM data. VEGTYPE was characterized using the CORINE Land Cover inventory (CLC 2012 v.18.5.1; http://land.copernicus.eu/pan-european/corine-land-cover/clc-2012). We combined the land cover classes into six categories: arable, urban‐associated, forest, rock‐associated, grass, and shrub (Table 1). Values of MINTEMP, PRECIPITATION, ALTITUDE, SLOPE, and ASPECT were calculated by taking the average value of each variable within a 100 m buffer around each sample location. In the case of VEGTYPE, the sample was assigned the category with the largest area within the buffer. ASPECT was classified as one of eight categories: N, NW, W, SW, S, SE, E, and NE.

**Table 1:** CORINE Land Cover (CLC 2012 v.18.5.1; http://land.copernicus.eu/pan-european/corine-land-cover/clc-2012) classes used to categorize vegetation type on Porto Santo

Vegetation type	CLC classes
Arable	Non‐irrigated arable land
	Vineyards
	Complex cultivation patterns
	Land principally occupied by agriculture with significant areas of natural vegetation
Urban‐associated	Discontinuous urban fabric
	Port areas
	Airports
	Sport and leisure (resort complex)
Forest	Coniferous forest
Rock‐associated	Beaches, dunes, and sand
	Bare rock
	Sparsely vegetated areas
Grass	Natural grassland
	Pastures
	Sport and leisure (golf course)
Shrub	Moors and heathland
	Transitional woodland‐shrub

We calculated DISTWATER with polygons drawn on Google Earth satellite imagery over water sources encountered during sample collection and obtained from the OpenStreetMap data‐filtering tool Overpass Turbo (https://overpass-turbo.eu) using the query “natural = water.” Water sources were 76–28,190 m^2^. The presence of livestock was dependent on visual encounters, as farming census data were not publicly available. The type of livestock was used to differentiate between factors related to livestock farming that might cause aggregations of birds (DISTFARM), and the potential effect of poultry as a reservoir of avian malaria (DISTPOUL). DIST_URB was the distance to the nearest urban‐associated area, as classified by VEGTYPE.

DENSITY was calculated as follows. A 1 km^2^ grid was overlaid on Porto Santo, and a 1 km radius buffer was drawn around the centroid of each grid cell. Any part of the buffer covering the ocean was removed. This was converted into a measure of pipits per km^2^ for each grid cell by dividing the number of samples within the buffer by its area. The average density within a 100 m buffer around each sample, weighted by the area of each 1 km^2^ grid cell occupied within the sample buffer, was used as the DENSITY measure.

### 2.7 Environmental predictors of malaria risk

We modeled the environmental predictors of malaria infection on Porto Santo and used the predicted values from this model as a measure of the malaria risk at each sample location. This allowed us to account for spatial variation in the likelihood of malaria exposure when analyzing the relationship between genotype and infection status. This was calculated using a model selection and then model averaging approach. Model selection tests all combinations of variables as predictors of the response variable and calculates the Akaike information criterion (AIC; Akaike, 1973) as a measure of fit of each model. Model averaging is then applied to a set of models with the lowest AIC (and therefore the highest likelihood) to calculate weighted averages of parameter estimates and the relative importance of model predictors (Burnham & Anderson, 2002). We report AICc, a modification of AIC that is recommended for small sample sizes (Hurvich & Tsai, 1989).

We used variance inflation factors (VIFs) calculated using the R package car (Fox & Weisberg, 2011) to test for collinearity between environmental variables, using a threshold of >3 to indicate unacceptably high collinearity (Zuur, Ieno, & Elphick, 2010). As we had categorical variables, we used generalized VIFs (GVIFs; Fox & Monette, 1992) transformed with (GVIF^1/2^ *^df^*)^2^ (*df* = degrees of freedom), to calculate a value equivalent to a standard VIF (Fox & Weisberg, 2011). When including all variables, every variable except ASPECT had VIFs > 3 (collinearity). We sequentially removed variables with VIFs > 3 that had the highest AICc scores from single‐predictor binomial generalized linear models (GLMs) of each variable against malaria infection status (Table 2). Variables were removed and VIFs recalculated until all variables had VIFs < 3. The remaining variables were VEGTYPE, ALTITUDE, DISTWATER, DENSITY, ASPECT, and DISTPOUL.

**Table 2:** Single‐predictor binomial generalized linear models of the environmental predictors of *Plasmodium* strain LK6 infection in adult Berthelot's pipits on Porto Santo

Variable	Estimate	*R* ^2^ a	*p*‐Value	AICc
ALTITUDE	−0.0160	0.129	<.001	100.0
DISTWATER	−0.0013	0.118	<.001	101.2
DIST_URB	−0.0014	0.113	<.001	101.8
VEGTYPE	—	0.157	.170	103.4
MINTEMP	2.5188	0.080	.005	105.4
SLOPE	−0.1569	0.069	.008	106.6
PRECIPITATION	−0.0308	0.041	.035	109.7
DISTPOUL	−0.0006	0.018	.152	112.2
DISTFARM	−0.0005	0.010	.284	113.1
DENSITY	0.0747	0.003	.586	113.9
ASPECT	—	0.094	.271	117.2

Note

Environmental variables are ordered by increasing AICc scores. Parameter estimates are not included for categorical variables.

a McFadden pseudo‐*R* ^2^.

Interactions between environmental variables may have biologically meaningful influences on malaria risk. For each biologically relevant pair of variables (Table 3), we tested whether the inclusion of an interaction term improved the fit of a binomial GLM with the two variables as main effects and malaria infection as the response. The interaction DENSITY*DISTWATER gave the largest improvement in AICc (main effects only, AICc = 103.3; main effects and interaction, AICc = 90.8) and was therefore included in model selection (Table 3).

**Table 3:** The effect of including biologically relevant interaction terms between environmental variables for predicting *Plasmodium* LK6 infection in adult Berthelot's pipits on Porto Santo

	Main effect AICc	Interaction AICc	ΔAICc	Retained
DENSITY*DISTWATER	103.3	90.8	−12.5	✓
DISTPOUL*DISTWATER	103.1	99.6	−3.5	
SLOPE*ALTITUDE	100.2	97.1	−3.1	
ALTITUDE*DISTWATER	92.3	91.2	−1.1	
ALTITUDE*DISTPOUL	101.2	101.3	0.1	
DENSITY*DISTPOUL	114.3	114.7	0.4	
ASPECT*SLOPE	116.4	118.3	1.9	
VEGTYPE*DENSITY	105.6	107.7	2.2	

Note

Binomial generalized linear models were performed with each pair of variables as main effects only, or including an interaction term. Where the addition of an interaction resulted in a change in AICc (ΔAICc) < −7, that interaction term was included in model selection.

### 2.8 Model selection and model averaging

Fitting all combinations of the six environmental variables and one interaction term (see above) as predictors of malaria infection using binomial GLMs, we performed model selection and model averaging following Grueber, Nakagawa, Laws, and Jamieson (2011) using the R package MuMIn (Bartoń, 2018) to obtain the best‐supported models for explaining occurrence of malaria infection. Prior to analysis, we used the R package arm (Gelman & Su, 2018) to standardize the input variables to a mean of zero and a standard deviation of 0.5 to enable meaningful comparisons of parameter estimates (Gelman, 2008; Grueber et al., 2011). The model selection process calculated ΔAICc, the difference in AICc between each model and the “best” model (the model with the lowest AICc), and the Akaike weight, which quantifies the likelihood of each model having the best explanatory power within a set of models (Burnham & Anderson, 2002). Using the R package DescTools (Signorell, 2018), we calculated the McFadden‐adjusted pseudo‐*R* ^2^ (the likelihood of a logistic regression model relative to an intercept‐only model, adjusted to account for the number of predictors in the model; McFadden, 1974). Values of McFadden *R* ^2^ between 0.2 and 0.4 represent a strong fit, equivalent to a linear regression *R* ^2^ of 0.7–0.9 (Louviere, Hensher, & Swait, 2000).

A threshold of ΔAICc ≤ 7 is recommended to retain models that have sufficient support, without dismissing models which still provide some explanatory power (Burnham, Anderson, & Huyvaert, 2011). We applied model averaging over this set of models to calculate weighted averages of parameter estimates and the relative importance of each predictor (the sum of Akaike weights for models which include that predictor). We used the zero method of model averaging to avoid biasing results toward predictors with low explanatory power (Burnham & Anderson, 2002; Lukacs et al., 2007).

### 2.9 Spatial autocorrelation

We tested for spatial autocorrelation in model residuals as this may lead to spurious associations between predictor and response variables (Dormann et al., 2007; Lennon, 2000). We created Moran's *I* correlograms at distance class intervals of 750 m and 1,000 m using the R package ncf (Bjornstad, 2018), with 1,000 permutations to test the significance of Moran's *I* at each interval. After correcting for multiple testing using the Holm correction (Holm, 1979; Legendre & Legendre, 2012), there was no evidence of spatial autocorrelation in the model residuals (all adjusted *p* values > .05). Correcting for spatial autocorrelation was therefore not required for the estimation of malaria risk in Porto Santo.

### 2.10 Malaria risk scores

We used the predicted values of the best model identified by model selection as a malaria risk score between 0 and 1 for each sample location. This represented the probability of an individual at that location being infected with malaria, as a result of the environmental conditions. An earlier study determined that malaria infection in Tenerife was best explained by DISTPOUL, DISTWATER, MINTEMP, and DISTWATER*MINTEMP (González‐Quevedo et al., 2014). As significant spatial autocorrelation was present in Tenerife, an autocovariate term was included in all model combinations during model selection, to account for autocorrelation up to 1,000 m (see González‐Quevedo et al., 2014). Hence, the predicted values from an autologistic model containing these predictors, interaction, and autocovariate were used as our estimate of malaria risk for Tenerife. Malaria risk was logit‐transformed prior to use in models.

### 2.11 Genetic associations with malaria infection

Genetic variation was classified in three ways: (a) SNP genotype, encoded as 1 for heterozygotes and 0 or 2 for each of the homozygotes; (b) presence (1) or absence (0) of each TLR4 protein haplotype; (c) SNP heterozygosity, with heterozygotes encoded as 1 and homozygotes as 0. Each model described below was performed three times, using each of the three classes of genetic variants as model predictors.

We first tested for associations between genetic variation and malaria infection status. We ran separate models for each island as the genetic divergence between Berthelot's pipits on Tenerife and Porto Santo, along with environmental differences in malaria risk patterns between the islands, might otherwise obscure genetic associations. We tested for genetic associations with malaria infection across all years using binomial generalized linear mixed models (GLMMs) with sampling year as a random effect, within Tenerife (2011) or Porto Santo (2016) using binomial GLMs. Testing across all years gave the advantage of a larger sample size; however, temporal fluctuations in selection pressures could interfere with genetic associations with malaria, so single‐year models were also performed.

We tested the genetic variables as predictors of malaria risk in general linear models (LMs) for Tenerife (2011) and Porto Santo (2016). As malaria risk was derived from spatially varying environmental predictors, genetic variation alone was unlikely to account for all spatial autocorrelation in malaria risk. We therefore included distance‐based Moran's eigenvector maps (dbMEMs) as spatial predictors of malaria risk. dbMEMs are used to identify gradients of spatial variation (spatial structure) in a response variable, across multiple potential scales from broad to fine, calculated by eigenvector decomposition of distance matrices based on the spatial coordinates of samples (Borcard & Legendre, 2002; Dray, Legendre, & Peres‐Neto, 2006). Hence, the use of dbMEMs as predictors of malaria risk accounts for spatially autocorrelated variation in malaria prevalence that would otherwise be explained by environmental conditions, transmission dynamics, and/or unmeasured genetic gradients. We calculated dbMEMs for each island using the R packages adespatial (Dray et al., 2018) and vegan (Oksanen et al., 2018), retaining dbMEMs with positive eigenvalues, representing positively autocorrelated spatial variation. dbMEMs were ranked by descending *R* ^2^ values in single‐predictor LMs of malaria risk and sequentially added into each model of the genetic associations with malaria risk outlined above, until additional dbMEMs no longer improved AICc. With each iteration, we checked whether spatial autocorrelation in model residuals had been controlled for, to find the minimum required number of dbMEMs. The inclusion of dbMEMs resulted in VIFs < 3, indicating that any collinearity between dbMEMs and genetic variants was acceptably low. We performed hierarchical partitioning using the “lmg” method in the R package relaimpo (Grömping, 2006) to calculate the proportion of variance in malaria risk explained by genetic variants.

## 3 RESULTS

### 3.1 Sequencing

Malaria was detected in 126 out of 190 individuals (66.3%) from Porto Santo and 189 out of 590 individuals (32.0%) from Tenerife (Table 4). All infected samples had one of four strains of *Plasmodium*, with no evidence of multiple infection. Between 2006 and 2010, only LK6 (Ortego et al., 2007) was detected (Illera et al., 2008; Spurgin et al., 2012). In samples from Tenerife in 2011, where *Plasmodium* was found in 148 of 388 individuals (38.1%), the majority of infections were LK6 (139 samples; 93.9%). Strains LK5 (Ortego et al., 2007) and KYS9 (Inci et al., 2012) were present in seven (4.7%) and two (1.4%) individuals, respectively (González‐Quevedo et al., 2014). Out of 129 samples collected from Porto Santo in 2016, 97 (75.2%) were infected. Of these, 91 (93.8%) had LK6, four (4.1%) had LK5, and a previously undocumented strain, PS1530, was found in two (2.1%) individuals. To avoid potential confounding factors arising from selection against different *Plasmodium* strains, we have focused here on the predominant LK6 strain.

**Table 4:** *Plasmodium* prevalence in Berthelot's pipits from Porto Santo and Tenerife across sampling years

Island	Year	*n*	Infected	*n* Adults	Infected adults	*n* Juveniles	Infected juveniles	LK6	LK5	KYS9	PS1530	*n* Final
Porto Santo		190	0.66	142	0.77	48	0.35	0.95	0.03	0.00	0.02	136
	2006	31	0.65	10	0.90	21	0.52	1.00	0.00	0.00	0.00	10
	2009	30	0.30	16	0.38	14	0.21	1.00	0.00	0.00	0.00	16
	2016	129	0.75	116	0.81	13	0.23	0.94	0.04	0.00	0.02	110
Tenerife		590	0.32	528	0.34	62	0.16	0.95	0.04	0.01	0.00	519
	2006	51	0.08	51	0.08	0	0.00	1.00	0.00	0.00	0.00	51
	2009	56	0.20	25	0.16	31	0.23	1.00	0.00	0.00	0.00	25
	2010	96	0.27	65	0.35	31	0.10	1.00	0.00	0.00	0.00	65
	2011	387	0.38	387	0.38	0	0.00	0.94	0.05	0.01	0.00	378

Note

Sample sizes are given for all birds (*n*), adults (*n* adults), and juveniles (*n* juveniles). The proportion of birds infected with any strain of *Plasmodium* is listed for all birds, adults, and juveniles. The different strains of *Plasmodium* found in Berthelot's pipits (LK6, LK5, KYS9, PS1530) are displayed as proportions of the infected samples. *n* final shows the sample size of the final dataset used in analyses of malaria risk and genetic associations after filtering out juveniles and samples infected with strains other than LK6.

Excluding sampling years where no juveniles were caught, the prevalence of malaria was significantly higher in adults than in juveniles, both in Porto Santo (test of equal proportions *χ* ^2^ = 25.6, *p* < 0.001) and in Tenerife (*χ* ^2^ = 3.1, *p* = 0.039). As juveniles were present in much lower numbers than adults (Table 4), we removed juveniles from further analysis. Final sample sizes are shown in Table 4.

**Figure 2:**
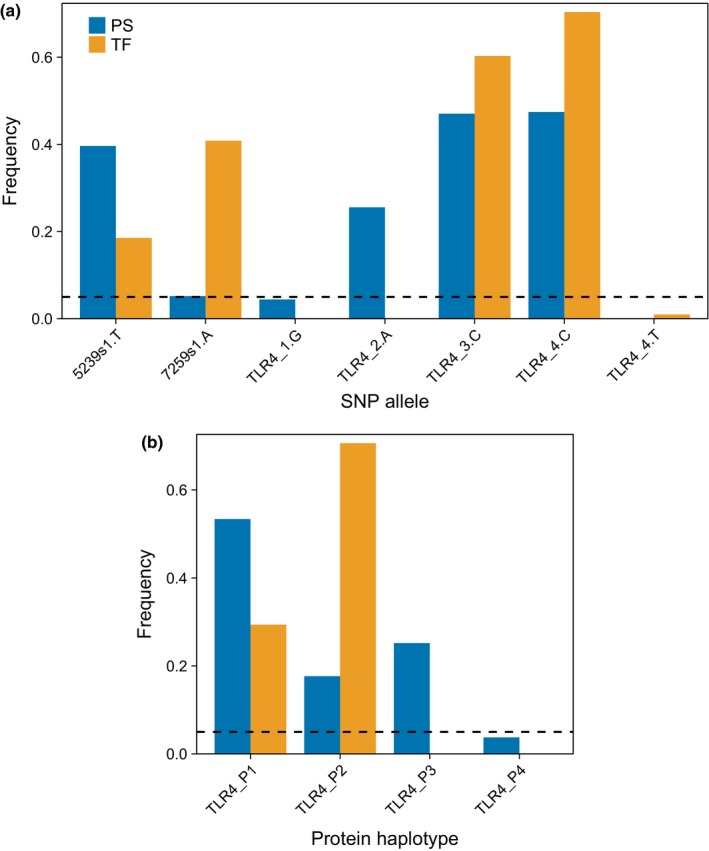
Genetic variant frequencies in adult Berthelot's pipits on Porto Santo (PS; blue) and Tenerife (TF; orange). (a) Allele frequencies per SNP. In each instance, the allele which is the minor allele in Porto Santo is represented. In addition, the low‐frequency T allele of the triallelic SNP TLR4_4 is also shown. (b) Frequencies of TLR4 protein alleles. In both plots, the black dashed line indicates a frequency threshold of 0.05; variants below this threshold were excluded from models of genetic associations with malaria risk and infection status

Sanger sequencing of TLR4 in the 2016 Porto Santo samples only detected SNPs which have been previously documented in Berthelot's pipits (González‐Quevedo et al., 2015). SNP allele frequencies differed between the two islands, with different minor alleles found at TLR4_3 and TLR4_4 (Figure 2a). Phasing of the TLR4 SNPs produced five nucleotide haplotypes (Table 5), all of which had been previously detected (González‐Quevedo et al., 2015). As amino acid substitutions could potentially alter TLR4 function (Schröder & Schumann, 2005), we classified TLR4 variation into four protein haplotypes (denoted with the prefix “TLR4_P”; Table 5 and Figure 2b). TLR4_P2 was translated from two haplotypes differing at the synonymous SNP TLR4_3. The TLR4_P3 and TLR4_P4 haplotypes were absent from Tenerife, and TLR4_P3 was at low frequency (<0.05) in Porto Santo.

**Table 5:** TLR4 nucleotide and protein haplotypes in Berthelot's pipits on Tenerife and Porto Santo

Haplotype	Sequence	Protein haplotype	Amino acid sequence
1	AGTA	TLR4_P1	DGPK
2	AGCC	TLR4_P2	DGPT
3	AGTC	TLR4_P2	DGPT
4	AACC	TLR4_P3	DDPT
5	GGCC	TLR4_P4	GGPT

We tested for deviations from Hardy–Weinberg equilibrium at each SNP for each combination of island and year, and in each island across all years. SNPs 5239s1 (Tenerife 2011 and all years), 7259s1 (Tenerife 2006 and 2009), and TLR4_1 (Porto Santo 2009) showed deviations from Hardy–Weinberg equilibrium at *p* < .05; however, following Holm correction for multiple comparisons, all adjusted *p* values were > 0.05. LD between SNPs TLR4_3 and TLR4_4 (excluding the low‐frequency T allele) was high (Porto Santo *R* ^2^ = 0.99; Tenerife *R* ^2^ = 0.62). We found moderate LD in Porto Santo between SNPs TLR4_2 and TLR4_3 (*R* ^2^ = 0.39) and between TLR4_2 and TLR4_4 (*R* ^2^ = 0.37). All other combinations of SNPs had low levels of LD (*R* ^2^ < 0.1).

### 3.2 Malaria risk models

**Figure 3:**
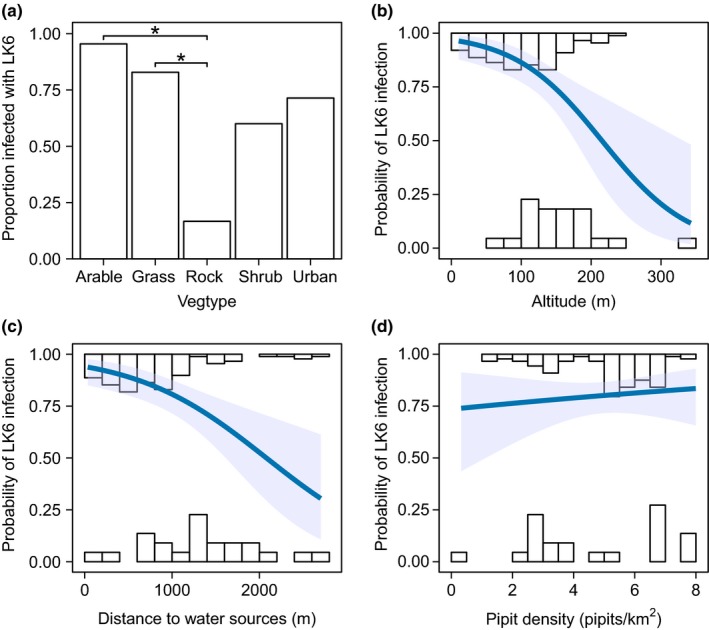
Environmental predictors of *Plasmodium* strain LK6 risk in adult Berthelot's pipits on Porto Santo. (a) The proportion of samples infected with LK6 per category of VEGTYPE. * indicates significant difference between categories in post hoc Tukey tests at *p* < 0.05. Plots b–d show logistic regression models of the effect of (b) altitude, (c) distance to water sources, and (d) pipit density on the probability of LK6 infection. Histograms indicate the frequency of uninfected (lower) and infected (upper) individuals for each (b) 50 m altitude class, (c) 200 m distance class, and (d) 0.5 pipits/km^2^ class. Shaded area represents 95% confidence intervals

**Figure 4:**
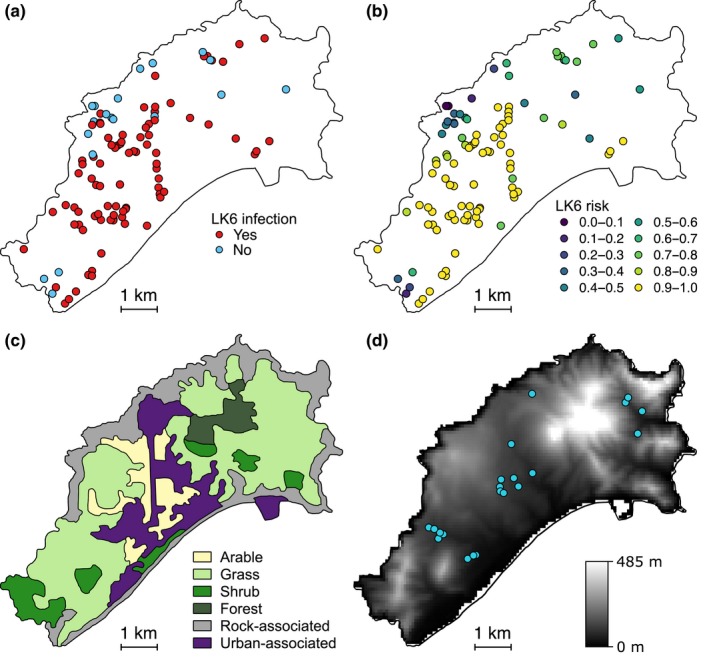
Spatial distribution of *Plasmodium* strain LK6 and environmental risk factors in adult Berthelot's pipits on Porto Santo. Points indicate location of (a) individual infection status and (b) infection risk in adult Berthelot's pipits. (c) VEGTYPE categories, adapted from the CORINE Land Cover inventory (CLC 2012 v.18.5.1; http://land.copernicus.eu/pan-european/corine-land-cover/clc-2012). (d) Altitude on Porto Santo, calculated from Shuttle Radar Topography Mission global elevation data (SRTM 90 m; https://srtm.csi.cgiar.org). Overlaid blue points indicate locations of standing water sources

**Figure 5:**
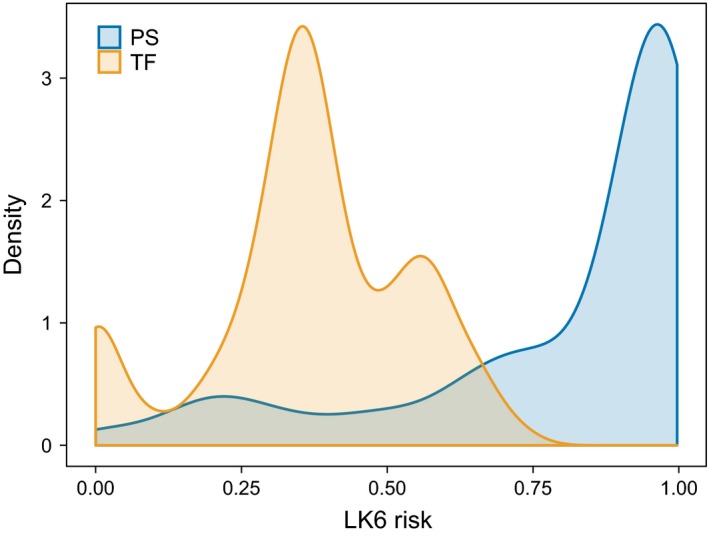
The distribution of *Plasmodium* strain LK6 infection risk in adult Berthelot's pipits on Porto Santo (PS; blue) and Tenerife (TF; orange)

Model selection of the environmental predictors of malaria infection found 17 models with ΔAICc ≤ 7 relative to the “best” model, which contained VEGTYPE, ALTITUDE, DISTWATER, DENSITY, and DISTWATER*DENSITY (Table 6). ALTITUDE and DISTWATER were negatively associated with malaria infection, whereas DENSITY was positively associated (Figure 3). A *post hoc* Tukey test of VEGTYPE as a predictor of malaria infection (using the R package multcomp; Hothorn, Bretz, & Westfall, 2008) found that rock‐associated habitat had a significantly negative effect on malaria infection relative to arable (*p* = 0.015) and grass habitats (*p* = 0.039; Figure 3). These four predictors, and the interaction term DISTWATER*DENSITY, had relative importances of 0.63–0.99 across the top model set (Table 7). We used the predicted values from the best model as our estimate for malaria risk for each sample location (Figure 4). DISTPOUL and SLOPE had low relative importance in model averaging (0.33 and 0.29, respectively), and ASPECT did not feature within the top model set. The malaria risk model for Porto Santo had a McFadden‐adjusted *R* ^2^ of 0.25 (values of 0.2–0.4 are equivalent to a linear regression *R* ^2^ of 0.7–0.9; Louviere et al., 2000), whereas the adjusted *R* ^2^ for the Tenerife malaria risk model was 0.10 (González‐Quevedo et al., 2014). Distributions of malaria risk differed markedly between the islands, with higher malaria risk in Porto Santo (Figure 5).

**Table 6:** Parameter estimates of predictors included in model selection of the environmental predictors of *Plasmodium* LK6 infection in adult Berthelot's pipits on Porto Santo

Model	Intercept	VEGTYPE	ALTITUDE	DENSITY	DISTPOUL	DISTWATER	SLOPE	DENSITY* DISTWATER	AICc	ΔAICc	Akaike weight	Adjusted *R* ^2^ a
1	2.62	+	−1.91	0.63		−1.54		−5.33	84.9	0.0	0.29	0.25
2	2.61	+	−1.75	0.40	−0.82	−1.39		−5.85	86.4	1.5	0.14	0.24
3	1.97		−1.32	1.01		−2.47		−4.17	86.4	1.5	0.13	0.22
4	2.63	+	−2.00	0.74		−1.56	0.31	−5.28	87.2	2.3	0.09	0.23
5	1.94		−1.23	0.79	−0.69	−2.18		−4.74	87.8	2.9	0.07	0.21
6	1.94		−1.08	0.83		−2.38	−0.52	−4.25	88.1	3.2	0.06	0.21
7	2.62	+	−1.96	0.59	−1.00	−1.34	0.70	−5.89	88.5	3.6	0.05	0.22
8	1.77			0.60		−2.36	−1.21	−4.79	89.0	4.1	0.04	0.20
9	1.93		−1.06	0.68	−0.62	−2.14	−0.42	−4.71	89.7	4.8	0.03	0.20
10	1.76			0.47	−0.65	−2.09	−1.05	−5.22	90.4	5.5	0.02	0.19
11	1.98	+		0.65		−1.81		−5.45	90.5	5.6	0.02	0.19
12	1.74			0.94		−2.43		−4.62	90.8	5.9	0.01	0.18
13	2.01	+		0.35	−1.09	−1.43		−6.20	91.0	6.1	0.01	0.19
14	1.72			0.66	−0.99	−2.04		−5.45	91.1	6.2	0.01	0.18
15	2.32	+	−2.45						91.3	6.4	0.01	0.18
16	2.06	+		0.36		−1.93	−0.98	−5.61	91.4	6.6	0.01	0.19
17	2.32	+	−2.12			−1.35			91.5	6.6	0.01	0.18

Note

Models within a threshold of ΔAICc ≤ 7 relative to the best model are shown.

a McFadden‐adjusted pseudo‐*R* ^2^.

**Table 7:** Environmental predictors of malaria risk in adult Berthelot's pipits on Porto Santo

	Best model	Model averaging	Relative importance
(Intercept)	2.62 (1.62, 4.04)	2.32 (1.10, 3.55)	
DISTWATER	−1.54 (−3.64, 0.67)	−1.79 (−3.83, 0.24)	0.99
DENSITY	0.63 (−0.59, 1.91)	0.66 (−0.64, 1.97)	0.98
DENSITY*DISTWATER	−5.33 (−9.01, −2.19)	−5.02 (−8.72, −1.32)	0.98
ALTITUDE	−1.91 (−3.85, −0.54)	−1.47 (−3.33, 0.39)	0.87
VEGTYPE	+	+	0.63
arable	−0.56 (−2.97, 2.58)	−0.31 (−2.42, 1.80)	
rock	−3.77 (−7.55, −1.10)	−2.42 (−6.91, 2.06)	
shrub	−3.37 (−7.28, −0.00)	−1.95 (−6.00, 2.11)	
urban	−1.59 (−3.85, 0.80)	−0.94 (−3.26, 1.38)	
DISTPOUL		−0.26 (−1.47, 0.94)	0.33
SLOPE		−0.05 (−1.27, 1.16)	0.29
Adjusted *R* ^2^ a	0.25		

Note

Parameter estimates (and 95% confidence intervals) are presented for the “best” model with lowest AICc as determined by model selection, and for model averaging across 17 models with ΔAICc ≤ 7 relative to the best model. Within VEGTYPE, coefficients of arable, rock, shrub, and urban were calculated relative to grass as the reference category.

a McFadden‐adjusted pseudo‐*R* ^2^.

### 3.3 Genetic associations with malaria infection

As we found high levels of LD between TLR4 SNPs, we calculated VIFs for models of genetic associations with malaria infection and risk. All VIF scores were <3 in Tenerife. In Porto Santo, TLR4_3 and TLR4_4 had elevated VIF scores (>50); however, after removing the synonymous TLR4_3 SNP, all VIF scores were <3.

**Figure 6:**
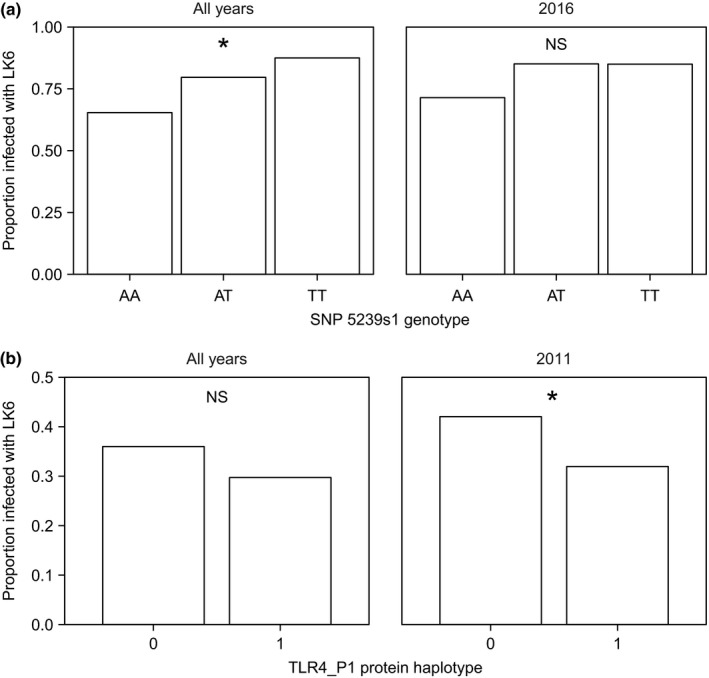
The association between *Plasmodium* strain LK6 infection status and genetic variants in adult Berthelot's pipits. (a) SNP 5239s1 genotype association with LK6 in Porto Santo. (b) Protein haplotype TLR4_P1 association with LK6 in Tenerife. 0 = absent, 1 = present. *Denotes significance at *p* < 0.05; NS = not significant

Results from models of genetic variants as predictors of malaria infection are summarized in Table 8. In Porto Santo, across all years, increasing frequency of the T allele in SNP 5239s1 was associated with increased malaria infection (estimate = 0.75, *SE* = 0.34, *p* = 0.026; Figure 6a). This effect was no longer significant when looking only at 2016 (*p* = 0.099), although the direction of the result remained consistent, with the highest probability of malaria prevalence in TT genotype individuals. This may be a power issue (*n* = 110 for 2016 vs. *n* = 136 for all years). No other SNP was significantly associated with malaria infection in 2016 or across all years. There were no associations between malaria infection and SNP heterozygosity or TLR4 protein haplotypes in Porto Santo.

**Table 8:** Summary of general linear models of the association between genetic variants and malaria infection status in Berthelot's pipits on Porto Santo (PS) and Tenerife (TF)

Island	Variant type	Variant	All years estimate	Main year estimate
PS	SNP genotype	5239s1	0.75 (0.34)*	0.60 (0.37)
		TLR4_2	0.90 (0.51)	1.10 (0.58)
		TLR4_4	0.15 (0.40)	−0.16 (0.45)
	SNP heterozygosity	5239s1	0.53 (0.45)	0.61 (0.52)
		TLR4_2	0.94 (0.49)	0.95 (0.55)
		TLR4_4	−0.76 (0.47)	−0.98 (0.53)
	TLR4 protein haplotype	TLR4_P1	−1.62 (0.85)	−1.57 (0.94)
		TLR4_P2	−0.53 (0.52)	−0.82 (0.60)
		TLR4_P3	0.40 (0.51)	0.24 (0.59)
TF	SNP genotype	5239s1	0.08 (0.17)	0.18 (0.18)
		7259s1	−0.02 (0.14)	−0.03 (0.15)
		TLR4_3	−0.10 (0.22)	0.09 (0.26)
		TLR4_4	0.31 (0.25)	0.19 (0.29)
	SNP heterozygosity	5239s1	0.09 (0.21)	0.25 (0.24)
		7259s1	−0.29 (0.20)	−0.30 (0.22)
		TLR4_3	0.20 (0.25)	0.00 (0.29)
		TLR4_4	−0.41 (0.26)	−0.40 (0.29)
	TLR4 protein haplotype	TLR4_P1	−0.34 (0.20)	−0.46 (0.23)*
		TLR4_P2	−0.07 (0.38)	−0.20 (0.43)

Note

Parameter estimates (with standard error in brackets) for each genetic variant were taken from multipredictor models with genetic variants coded as SNP heterozygosity, SNP genotype, or TLR4 protein haplotype presence/absence. Models were performed in each island across all sampling years (“All years estimate”), or in the main sampling year with largest sample size (Porto Santo = 2016; Tenerife = 2011; “Main year estimate”). Asterisks next to parameter estimates denote significance of the predictor (*p < 0.05).

TLR4_P1 presence had a negative effect on malaria infection on Tenerife in 2011 (estimate = −0.46, *SE* = 0.23, *p* = 0.041; Figure 6b). We found the same trend across all years, although the association was not significant (*p* = 0.091). The TLR4_P2 haplotype was not associated with malaria infection in 2011 or across all years. We found no associations with malaria infection for SNP genotypes or SNP heterozygosity in Tenerife.

### 3.4 Genetic associations with malaria risk

**Figure 7:**
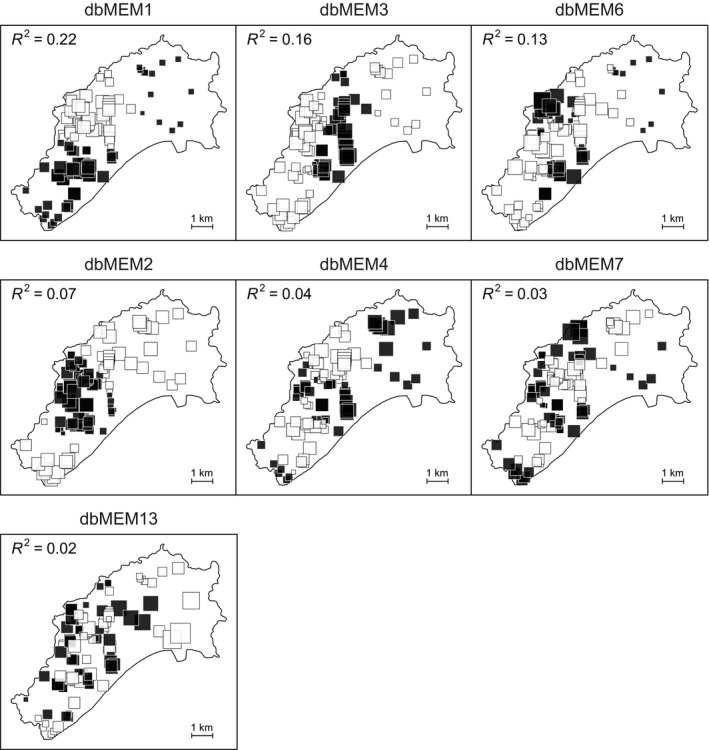
Distance‐based Moran's eigenvector maps (dbMEMs) showing strongest associations with *Plasmodium* strain LK6 infection risk in adult Berthelot's pipits on Porto Santo. *R* ^2^ values for each dbMEM were calculated from single‐predictor LMs of each dbMEM against logit‐transformed LK6 risk. Positive eigenvector scores are indicated by black squares, and negative scores are white. The size of the square indicates the magnitude of the score

**Figure 8:**
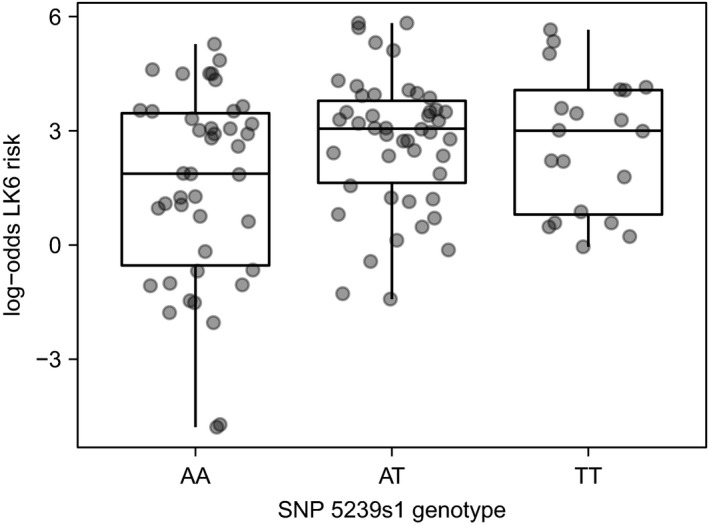
The association between *Plasmodium* strain LK6 infection risk and SNP 5239s1 in adult Berthelot's pipits on Porto Santo. LK6 risk was logit‐transformed prior to model fitting; a log‐odds risk score of 0 is equivalent to an infection probability of .5

We tested for associations between genetic variants and malaria risk on Porto Santo (2016) and Tenerife (2011). The results are summarized in Table 9. On Porto Santo, increasing numbers of T alleles at SNP 5239s1 (estimate = 0.69, *SE* = 0.27, *p* = 0.011), and A alleles at SNP TLR4_2 (estimate = 1.03, *SE* = 0.42, *p* = 0.016), were associated with increased malaria risk. However, the residuals of this model were highly spatially autocorrelated. To control for this, we included seven dbMEMs with high *R* ^2^ in single‐predictor models of malaria risk (Figure 7), chosen from a set of dbMEMs which gave the lowest AICc in a multipredictor model of malaria risk. After controlling for autocorrelation, SNP 5239s1 was still associated with malaria risk (estimate = 0.38, *SE* = 0.17, *p* = 0.030; Figure 8) but TLR4_2 was not (*p* = 0.423). We did not find an association between TLR4_4 and malaria risk, either before or after controlling for autocorrelation. Hierarchical partitioning of the above models (Table 10) found that SNP 5239s1 explained 5.2% of the variance in malaria risk before controlling for autocorrelation, and 3.3% of the variance after the addition of dbMEMs. Despite having a non‐significant association with malaria risk in the model containing dbMEMs, TLR4_2 explained a greater proportion of the variance in malaria risk compared to 5239s1, both before (7.4%) and after (4.3%) controlling for autocorrelation.

**Table 9:** Summary of linear models of the association between genetic variants and malaria risk in Berthelot's pipits on Porto Santo (PS) and Tenerife (TF)

Island	Variant type	Variant	Estimate	dbMEM estimate
PS	SNP genotype	5239s1	0.69 (0.27)*	0.38 (0.17)*
		TLR4_2	1.03 (0.42)*	0.22 (0.27)
		TLR4_4	0.21 (0.36)	0.09 (0.23)
	SNP heterozygosity	5239s1	0.79 (0.41)	0.74 (0.25)**
		TLR4_2	0.77 (0.42)	0.39 (0.26)
		TLR4_4	−0.59 (0.42)	−0.36 (0.26)
	TLR4 protein haplotype	TLR4_P1	−1.32 (0.62)*	−0.41 (0.41)
		TLR4_P2	−0.36 (0.51)	0.02 (0.33)
		TLR4_P3	0.60 (0.47)	0.14 (0.30)
TF	SNP genotype	5239s1	0.26 (0.73)	
		7259s1	−0.23 (0.60)	
		TLR4_3	−0.03 (1.04)	
		TLR4_4	0.40 (1.13)	
	SNP heterozygosity	5239s1	0.80 (0.96)	
		7259s1	−1.08 (0.86)	
		TLR4_3	0.48 (1.13)	
		TLR4_4	−0.59 (1.14)	
	TLR4 protein haplotype	TLR4_P1	−0.45 (0.89)	
		TLR4_P2	0.22 (1.68)	

Note

Parameter estimates (with standard error in brackets) for each genetic variant were taken from multipredictor models with genetic variants coded as SNP heterozygosity, SNP genotype, or TLR4 protein haplotype presence/absence. On Porto Santo, models were performed with just the genetic variants, or with the inclusion of dbMEMs to control for autocorrelation in model residuals (“dbMEM estimate”). dbMEMs were unable to account for autocorrelation in Tenerife models. Asterisks next to parameter estimates denote significance of the predictor (*p <0 .05; **p < 0.01).

**Table 10:** Hierarchical partitioning of variance for predictors of malaria risk in Berthelot's pipits on Porto Santo

Predictor	*R* ^2^	*R* ^2^ (with dbMEMs)
5239s1	0.052	0.033
TLR4_2	0.074	0.043
TLR4_4	0.039	0.027
dbMEMs		0.589

Note

*R* ^2^ values were calculated from a general linear model with genotypes at SNPs 5239s1, TLR4_2, and TLR4_4 as predictors of malaria risk, with and without the inclusion of dbMEMs as predictors to control for autocorrelation.

Before taking autocorrelation into account, there were no significant associations between SNP heterozygosity and malaria risk on Porto Santo; however, after including seven dbMEMs to remove spatial autocorrelation in model residuals, heterozygosity at SNP 5239s1 was strongly associated with increased malaria risk (estimate = 0.74, *SE* = 0.25, *p* = 0.004). Heterozygosity at other SNPs was not associated with malaria risk.

Prior to controlling for autocorrelation, the presence of protein haplotype TLR4_P1 was associated with reduced malaria risk on Porto Santo (estimate = −1.32, *SE* = 0.62, *p* = 0.037). The same seven dbMEMs were used to control for spatial autocorrelation, after which there was no longer an effect of TLR4_P1 presence (*p* = 0.318). No other TLR4 protein haplotypes showed associations with malaria risk.

To investigate the loss of significance of the associations between TLR4_P1 or TLR4_2 and malaria risk on Porto Santo, we ran a binomial GLM (TLR4_P1) and LM (TLR4_2) with the seven dbMEMs as predictors for the two genetic variants. dbMEM1 was significantly associated with both TLR4_P1 (estimate = −1.18, *SE* = 0.39, *p* = 0.003) and TLR4_2 (estimate = 0.16, *SE* = 0.06, *p* = 0.005).

We did not find any significant associations between SNP genotype, SNP heterozygosity, or TLR4 protein haplotypes and malaria risk on Tenerife. We were unable to remove spatial autocorrelation in model residuals through the addition of dbMEMs as model predictors.

## 4 DISCUSSION

We used previously identified candidate SNPs linked to malaria infection across populations (from a GWAS analysis performed on RAD‐seq SNPs; Armstrong et al., 2018) and TLR4 SNPs (González‐Quevedo et al., 2015) to investigate the relationship between potentially adaptive genetic variation and avian malaria within two island populations of Berthelot's pipits. In addition to testing for associations with infection status, we calculated the malaria risk at each sampling location, predicted by modeling fine‐scale environmental drivers of malaria infection. We found associations between malaria infection status and SNP 5239s1 in Porto Santo, and TLR4 protein haplotype 1 in Tenerife. Furthermore, the SNPs 5239s1 and TLR4_2 showed associations with malaria risk in Porto Santo, but not in Tenerife, where malaria risk was lower.

### 4.1 Genetic associations with malaria

We have previously used RAD‐seq SNPs to detect genetic variants that were associated with LK6 infection in Berthelot's pipits in the Canary Islands (Armstrong et al., 2018). The strongest association was found for SNP 5239s1, ca. 2,000 bp from interleukin‐16, a proinflammatory cytokine that moderates the expression of other cytokines associated with malaria infection (Kern, Hemmer, Damme, Gruss, & Dietrich, 1989; Lyke et al., 2004; Mathy et al., 2000). In the present study, SNP 5239s1 was a predictor of malaria on Porto Santo, with the lowest infection and risk found in samples with the AA genotype. Remarkably, this was the opposite relationship to that found in the Canary Islands (Armstrong et al., 2018), where increased incidence of the T allele was associated with reduced infection. This may be indicative of pathogen‐mediated balancing selection, which can arise from heterozygote advantage (Doherty & Zinkernagel, 1975), rare‐allele advantage (Slade & McCallum, 1992; Takahata & Nei, 1990), and local adaptation to fluctuating pathogen selection pressures (Hill et al., 1991). When controlling for spatial autocorrelation, we found an association between SNP 5239s1 heterozygosity and malaria risk on Porto Santo, although contrary to the heterozygote advantage model, heterozygotes were associated with greater malaria risk than homozygotes (an effect which was largely driven by the decline in risk found with AA genotypes). Berthelot's pipit populations on the Madeiran and Canary Islands archipelagos have been isolated from each other for at least 8,500 years (Spurgin et al., 2014). Different populations may therefore be undergoing independent coevolutionary cycles with the same malaria strain, with alternative alleles conferring an advantage between divergent populations (Bonneaud, Pérez‐Tris, Federici, Chastel, & Sorci, 2006). Alternatively, undetected genetic and phenotypic differences within the LK6 strain could potentially drive local adaptation between the archipelagos, with different alleles favored in different populations (Alcaide, Edwards, Negro, Serrano, & Tella, 2008; Loiseau et al., 2009). We used a single genetic marker, the mitochondrial cytochrome b locus, to classify the malaria strain. Several genes on the *Plasmodium* genome with relevance to infection success have shown greater genetic variation than at cytochrome b (Jarvi, Farias, & Atkinson, 2008; Lauron et al., 2014). It is possible that Berthelot's pipits on separate archipelagos could be adapting to different malaria strains within LK6, although this remains to be tested.

We did not find evidence of associations between SNP 5239s1 and malaria infection or risk on Tenerife, despite this population being included in the previous GWAS (Armstrong et al., 2018). SNPs that are related to individual‐level variation in parasite burden do not necessarily show the same associations at the landscape scale (Wenzel, Douglas, James, Redpath, & Piertney, 2016). It is possible that with the comparatively low malaria risk in Tenerife, gene flow is overriding landscape‐scale associations between SNP 5239s1 and malaria risk (Forester, Jones, Joost, Landguth, & Lasky, 2016; Lenormand, 2002). The previous GWAS result could have been driven by other populations such as Lanzarote and Fuerteventura, where malaria infection rates were higher (Illera et al., 2008; Spurgin et al., 2012).

Polymorphisms in immune genes can alter the effectiveness of their proteins for detecting and responding to pathogens (Lazarus et al., 2002; Sommer, 2005). The TLR4 SNPs sequenced here are situated within the ligand‐binding region, which plays a key role in TLR pathogen recognition (Werling, Jann, Offord, Glass, & Coffey, 2009). Evidence of positive selection in birds or mammals has been detected at each of the codons identified as polymorphic in Berthelot's pipits (Areal, Abrantes, & Esteves, 2011; Králová et al., 2018; Wlasiuk & Nachman, 2010), suggesting that these sites may be important for the evolution of pathogen recognition. On Tenerife, the presence of the TLR4 protein haplotype TLR4_P1 was associated with decreased malaria infection prevalence in 2011, but not across all sampling years. In earlier years, approximately half of the samples were collected from the high‐altitude (>2,000 m above sea level) plateau of El Teide. Malaria has not been found in Berthelot's pipits in this location (González‐Quevedo et al., 2014; Illera et al., 2008; Spurgin et al., 2012), although a survey of passerine communities on Tenerife found malaria at low frequency in high‐altitude habitats (Padilla et al., 2017). The relationship seen in 2011 between TLR4_P1 and infection may be masked in other sampling years by the increase in uninfected individuals from areas of low malaria abundance. We did not find a relationship between TLR4_P1 presence and malaria risk, potentially due to the explanatory power of the Tenerife malaria risk model (McFadden‐adjusted pseudo‐*R* ^2^ = 0.10). On Porto Santo, both TLR4_P1 and the SNP TLR4_2 were associated with malaria risk, although these relationships were no longer significant after including dbMEMs to remove autocorrelation. Both of these genetic variants showed significant associations with dbMEM1, which itself explained 22% of the variance in malaria risk, making it difficult to disentangle the real effects of these variants from any spurious associations arising from residual autocorrelation.

### 4.2 Environmental predictors of malaria risk

We modeled the environmental predictors of malaria distributions in Porto Santo to understand fine‐scale spatial differences in malaria risk. Higher altitudes were associated with decreased probability of malaria infection on Porto Santo, whereas on Tenerife, temperature was a predictor of malaria. Collinearity between altitude, temperature, and precipitation was found on both islands, with the same climatic processes likely influencing malaria distributions (González‐Quevedo et al., 2014). This is perhaps not surprising as malaria vector distributions are constrained by thermal requirements, with decreased malaria prevalence often reported at high altitudes (Eggert et al., 2008; Niebuhr, Poulin, & Tompkins, 2016) and low temperatures (Blanford et al., 2013; Craig, Le Sueur, & Snow, 1999; Loiseau et al., 2013).

Distance to water sources was an important predictor of malaria distributions in both Porto Santo and Tenerife (González‐Quevedo et al., 2014). Due to the aquatic larval development of mosquitoes, higher vector abundance and malaria are found in proximity to water (Ferraguti et al., 2018; Ganser et al., 2016; Illera et al., 2017). In the present study, distance to urban areas was removed prior to model selection due to a positive collinearity with distance to water sources. Therefore, we cannot rule out the importance of additional sources of standing water that may be associated with urban environments. Other studies have found links between urbanization and increased malaria and/or vector abundance (Alemu, Tsegaye, Golassa, & Abebe, 2011; Li et al., 2014), although this appears to vary between vector species, with some favoring more natural habitats (Ferraguti et al., 2016). Pipit density was positively associated with malaria risk on Porto Santo, although the model‐averaged parameter estimate was relatively small. There was, however, a strong negative interaction between distance to water and pipit density on this island, likely due to aggregations of mosquitoes and hosts around water sources, which may increase disease transmission rates (Begon et al., 2002; Greer, Briggs, & Collins, 2008; Le Menach, McKenzie, Flahault, & Smith, 2005; Raghwani et al., 2011).

Vegetation type was associated with malaria prevalence on Porto Santo. The highest abundance of malaria was found in arable and grassland habitats, with lower malaria in rock‐associated habitats. However, this result should be interpreted with caution due to small sample sizes, as only six pipits were caught on rock‐associated habitats. While not an important predictor of malaria infection in Tenerife (González‐Quevedo et al., 2014), differences in malaria and vector abundances between vegetation types have been found elsewhere (Clark, Wells, Dimitrov, & Clegg, 2016; Ferreira Junior et al., 2017; Rubio‐Palis & Zimmerman, 1997).

Contrary to findings from Tenerife (González‐Quevedo et al., 2014), distance to poultry was not an important predictor of malaria prevalence on Porto Santo. This may be because the effects of poultry farms as disease reservoirs (either due to the poultry themselves or due to aggregations of wild birds around them), that are driving increased malaria abundance on Tenerife (González‐Quevedo et al., 2014), do not have an effect at the small scale of poultry farming witnessed on Porto Santo.

By testing for associations with malaria infection and risk at TLR4 and novel malaria‐associated SNPs in divergent populations, we have revealed contrasting patterns of malaria risk and potential local adaptation, potentially due to different patterns of coevolution between the two populations. In addition, we found genetic associations with environmentally driven fine‐scale spatial variation in malaria risk at the landscape scale within Porto Santo. A lack of genetic associations with malaria risk in Tenerife may indicate the importance of spatial scales for assessing local adaptation across landscapes, where fine‐scale associations may be obscured over larger areas. Understanding the processes of local adaptation and the environmental drivers of infectious disease will be of additional importance for conservation efforts, as future climatic fluctuations alter the prevalence of disease.

## CONFLICT OF INTEREST

None declared.

## AUTHOR CONTRIBUTIONS

DSR, RGD, and LGS designed the research, obtained funding, and supervised the project. DSR, LGS, CG‐Q, and CA collected samples. CA, CG‐Q, and MD performed laboratory work and analyzed sequences. Spatial analyses were devised by CA, CG‐Q, and RGD and undertaken by CA and CG‐Q with input from DSR, RGD, and LGS. CA drafted the manuscript, with input from DSR, RGD, and LGS. All authors contributed critically to the drafts and approved the final manuscript for publication.

## Data Availability

PS1530 Plasmodium sequence: GenBank accession MN434072. Sample, genotype, and environmental data: Dryad https://doi.org/10.5061/dryad.228986b.
